# New Aldehyde‐Functional Methacrylic Water‐Soluble Polymers

**DOI:** 10.1002/anie.202015298

**Published:** 2021-05-03

**Authors:** Emma E. Brotherton, Craig P. Jesson, Nicholas J. Warren, Mark J. Smallridge, Steven P. Armes

**Affiliations:** ^1^ Chemistry The University of Sheffield Dainton Building, Brook Hill Sheffield S3 7HF UK; ^2^ GEO Specialty Chemicals Charleston Road, Hardley, Hythe Southampton SO45 3ZG UK

**Keywords:** aldehyde-functional methacrylic monomers, block copolymers, periodate oxidation, RAFT polymerization

## Abstract

Aldehyde groups enable facile conjugation to proteins, enzymes, oligonucleotides or fluorescent dyes, yet there are no literature examples of water‐soluble aldehyde‐functional vinyl monomers. We report the synthesis of a new hydrophilic *cis*‐diol‐based methacrylic monomer (GEO5MA) by transesterification of isopropylideneglycerol penta(ethylene glycol) using methyl methacrylate followed by acetone deprotection via acid hydrolysis. The corresponding water‐soluble aldehyde monomer, AGEO5MA, is prepared by aqueous periodate oxidation of GEO5MA at 22 °C. RAFT polymerization of GEO5MA yields the water‐soluble homopolymer, PGEO5MA. Aqueous periodate oxidation of the terminal *cis*‐diol units on PGEO5MA at 22 °C affords a water‐soluble aldehyde‐functional homopolymer (PAGEO5MA). Moreover, a library of hydrophilic statistical copolymers bearing *cis*‐diol and aldehyde groups was prepared using sub‐stoichiometric periodate/*cis*‐diol molar ratios. The aldehyde groups on PAGEO5MA homopolymer were reacted in turn with three amino acids to demonstrate synthetic utility.

## Graphical abstract

A water‐soluble aldehyde monomer (AGEO5MA) was prepared by oxidation of GEO5MA at 22 °C. RAFT aqueous polymerization yielded PAGEO5MA homopolymer, which was oxidized using sub‐stoichiometric periodate/*cis*‐diol molar ratios to yield hydrophilic statistical copolymers with *cis*‐diol and aldehyde groups. Three PGEO5MA‐based diblock copolymers were converted into aldehyde‐functional copolymers, which were derivatized with amino acids.

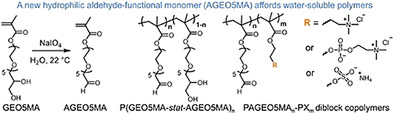

## Introduction

Aldehydes are extremely useful functional groups in synthetic organic chemistry: they can be oxidized to give carboxylic acids, reduced to afford alcohols, undergo Schiff base chemistry and also form (hemi)acetals.^[1]^ In the field of synthetic polymer chemistry, aldehyde‐based initiators[^2^, ^3^, ^4^, ^5^, ^6^, ^7^, ^8^] have been utilized to prepare various types of aldehyde‐functional polymers. Alternatively, Bilgic and Klok derivatized poly(2‐hydroxyethyl methacrylate) brushes under oxidative conditions in order to introduce aldehyde groups for subsequent oligonucleotide conjugation.^[9]^ However, surprisingly few aldehyde‐functional monomers have been reported in the literature.[^10^, ^11^, ^12^, ^13^, ^14^, ^15^, ^16^, ^17^, ^18^, ^19^, ^20^, ^21^, ^22^, ^23^, ^24^, ^25^] Most of these examples are hydrophobic (e.g. 4‐vinylbenzaldehyde) and hence produce water‐insoluble polymers.[^16^, ^19^, ^20^, ^21^, ^22^, ^23^, ^26^, ^27^, ^28^, ^29^, ^30^, ^31^, ^32^] This is unfortunate, because aldehyde groups enable facile conjugation to peptides/proteins and water‐soluble dyes in aqueous solution under mild conditions.[^2^, ^3^, ^4^, ^5^, ^10^, ^25^, ^33^, ^34^, ^35^, ^36^, ^37^, ^38^] In principle, this problem can be circumvented by statistical copolymerization of the hydrophobic aldehyde‐functional monomer with a sufficiently hydrophilic comonomer.[^12^, ^13^, ^15^, ^25^, ^33^, ^39^] Alternatively, the incorporation of a terminal protected aldehyde moiety onto a poly(ethylene glycol) (PEG) chain has been utilized to confer aldehyde functionality under aqueous conditions.[^5^, ^6^, ^7^, ^8^, ^40^, ^41^] Nevertheless, despite the remarkable progress made in synthetic polymer chemistry over the past few decades, there seem to be few, if any, literature examples of hydrophilic aldehyde‐functional vinyl monomers and their corresponding water‐soluble homopolymers.

**Scheme 1:**
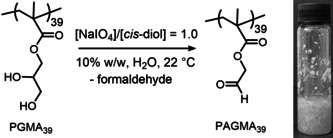
Selective oxidation of a water‐soluble PGMA_39_ homopolymer precursor using a stoichiometric amount of sodium periodate in aqueous solution at 22 °C affords PAGMA_39_ as a water‐insoluble precipitate.

One well‐known route to aldehyde‐terminated water‐soluble polymers is the selective oxidation of the minor fraction of *cis*‐diol units within poly(vinyl alcohol).^[42]^ This water‐soluble polymer can be obtained via hydrolysis of poly(vinyl acetate), which contains such *cis*‐diols as defect sites resulting from a small amount of head‐to‐head coupling during the free radical homopolymerization of vinyl acetate.^[43]^ Oxidation is readily achieved in aqueous solution under mild conditions using sodium periodate to afford aldehyde‐capped poly(vinyl alcohol) chains.^[44]^ Inspired by this well‐established chemistry, we recently decided to investigate the periodate oxidation of poly(glycerol monomethacrylate) (PGMA) to produce an aldehyde‐functional methacrylic polymer (Scheme 1; Supporting Information). However, periodate oxidation of a 10 % w/w aqueous solution of PGMA_39_ at 22 °C merely produced a macroscopic precipitate. This suggests that the target aldehyde‐functional methacrylic homopolymer (PAGMA) is actually hydrophobic. In principle, such precipitation could be the result of reaction between the *cis*‐diol and aldehyde units at intermediate conversion. However, reaction exotherms (data not shown) and visual inspection of the reaction mixtures suggest that the timescale required for the *cis*‐diol oxidation is much shorter than that for precipitation. Thus, it seems more likely that intermolecular crosslinking occurs between geminal diols and aldehydes (Supporting Information, Scheme S1).

**Scheme 2:**
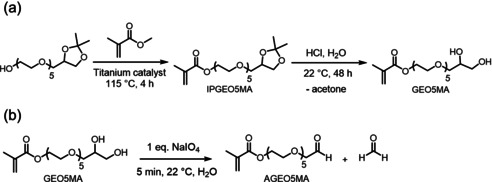
a) Two‐step synthesis of GEO5MA monomer starting from an isopropylidene glycerol precursor as a hydroxy‐functional initiator. This precursor is then transesterified with methyl methacrylate to produce IPGEO5MA, before removing the ketal protecting group with acid to afford GEO5MA monomer. b) Oxidation of GEO5MA in aqueous solution using sodium periodate at 22 °C affords AGEO5MA with formaldehyde as a by‐product. The same selective oxidation can be used to convert PGEO5MA homopolymer into PAGEO5MA homopolymer using identical reaction conditions.

In view of these problems, we designed a new *cis*‐diol‐based methacrylic monomer (GEO5MA; Scheme 2 a). We envisaged that the pendent oligo(ethylene glycol) moiety in GEO5MA should confer sufficient hydrophilic character to ensure water solubility after converting its terminal *cis*‐diol group into an aldehyde via periodate oxidation to form either AGEO5MA monomer (Scheme 2 b) or the corresponding PAGEO5MA homopolymer.

## Results and Discussion

**Figure 1:**
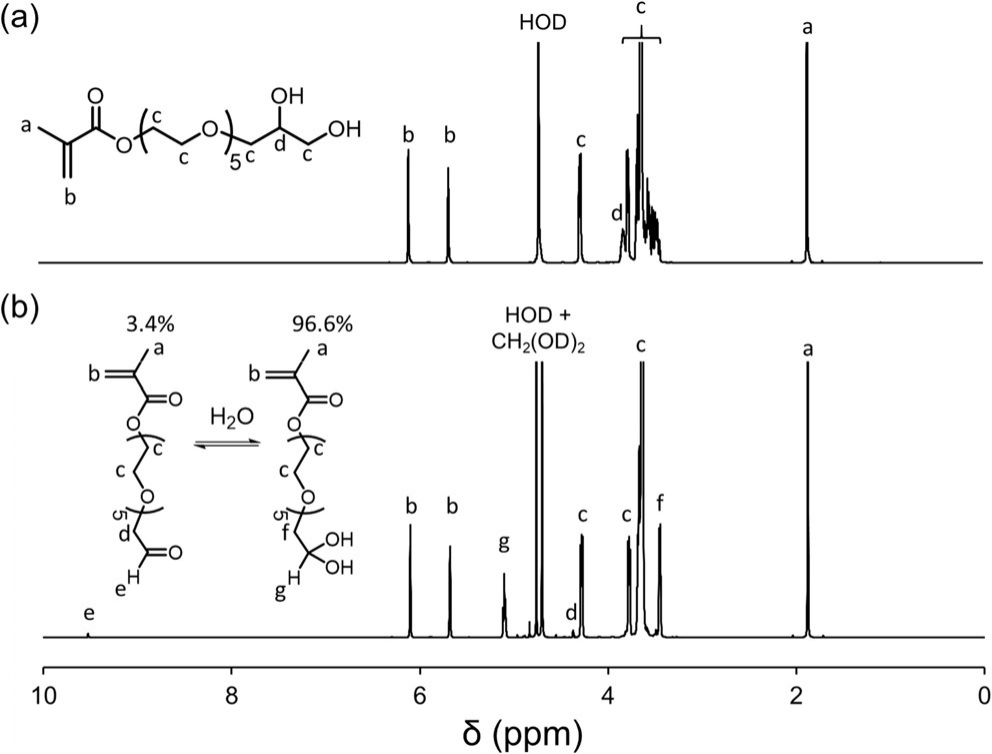
^1^H NMR spectra (D_2_O) recorded for a) GEO5MA monomer and b) AGEO5MA monomer (CH_2_(OD)_2_ denotes the hydrated form of formaldehyde).

The two‐step synthesis of GEO5MA monomer was conducted on a 1.2 kg scale via 1) transesterification of isopropylideneglycerol penta(ethylene glycol) using methyl methacrylate to afford IPGEO5MA (Scheme 2 a) and 2) acid hydrolysis to remove the acetone protecting group (Supporting Information). The chemical structure of this new methacrylic monomer was confirmed by ^1^H and ^13^C NMR spectroscopy (Figure 1 a; Supporting Information, Figure S1a), mass spectrometry, elemental microanalysis and FT‐IR spectroscopy (Supporting Information). The integrated signals in the ^1^H NMR spectrum are consistent with the proposed monomer structure. Its ^13^C NMR spectrum contained ten distinct signals. A characteristic signal at ≈160 ppm was assigned to the ester carbonyl carbon; its relatively low intensity is attributed to the slow relaxation time for such quaternary carbon atoms.^[45]^ The presence of a methacrylate group is confirmed by signals at 135 and 127 ppm. Several signals between 62.6 and 71.3 ppm are assigned to the pendent oligo(ethylene glycol) chain and include characteristic signals for the carbons attached to hydroxyl groups. According to mass spectrometry, the number of ethylene glycol units per oligo(ethylene glycol) group ranged from 2 to 7, with a mean value of 5.

**Figure 2:**
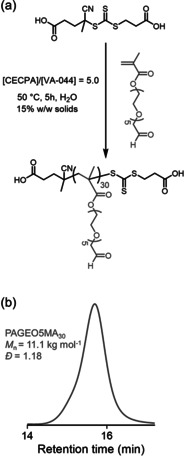
a) Synthesis of PAGEO5MA_30_ via RAFT aqueous solution polymerization of AGEO5MA using a water‐soluble dicarboxylic acid‐functionalized RAFT agent (CECPA). b) DMF GPC trace for the resulting PAGEO5MA_30_ homopolymer (molecular weight data expressed relative to poly(methyl methacrylate) calibration standards).

Oxidation of a 10 % w/w aqueous solution of GEO5MA using a NaIO_4_/*cis*‐diol molar ratio of unity (Scheme 2 b) led to essentially complete oxidation of the terminal *cis*‐diol units within 5 min at 22 °C, as confirmed by ^1^H NMR spectroscopy (Figure 1). The structure of this new AGEO5MA monomer was confirmed by mass spectrometry, elemental microanalysis, ^1^H and ^13^C NMR (Figure 1 b; Figure S1b) and FT‐IR spectroscopy (Figure S3a). Two new signals appear at 9.52 and 5.09 ppm in the ^1^H NMR spectrum for AGEO5MA, corresponding to an aldehyde group and a geminal diol, respectively. The aldehyde/geminal diol molar ratio was 0.034, which indicates that AGEO5MA exists primarily in its hydrated geminal diol form in D_2_O (Figure 1 b). Similar observations have been reported for other hydrophilic aldehydes in aqueous solution, such as acetaldehyde (Figure S2).[^46^, ^47^, ^48^, ^49^] During the periodate oxidation of GEO5MA to form AGEO5MA, the starting material can in principle react with the product to generate dimethacrylate species via (hemi)acetal chemistry.^[1]^ In practice, the final product contains less than 1 % dimethacrylate impurity as estimated by ^1^H NMR spectroscopy. The ^13^C NMR spectrum also shows the appearance of two new signals at 169.5 and 88.0 ppm, which correspond to the aldehyde carbon and the geminal diol carbon, respectively. After purification by extraction with CH_2_Cl_2_, the RAFT aqueous solution polymerization of AGEO5MA was conducted using a dicarboxylic acid‐functionalized water‐soluble RAFT agent (CECPA) to target a mean degree of polymerization (DP) of 30 (Figure 2 a). More than 99 % conversion was achieved and the resulting PAGEO5MA_30_ was well‐defined, as indicated by its relatively narrow, unimodal GPC trace (*M*_n_=11 100 g mol^−1^; *Đ*=1.18; Figure 2 b). ^1^H NMR signals for the terminal aldehyde and geminal diol groups were detected for this homopolymer (aldehyde/geminal diol molar ratio=0.041).

**Figure 3:**
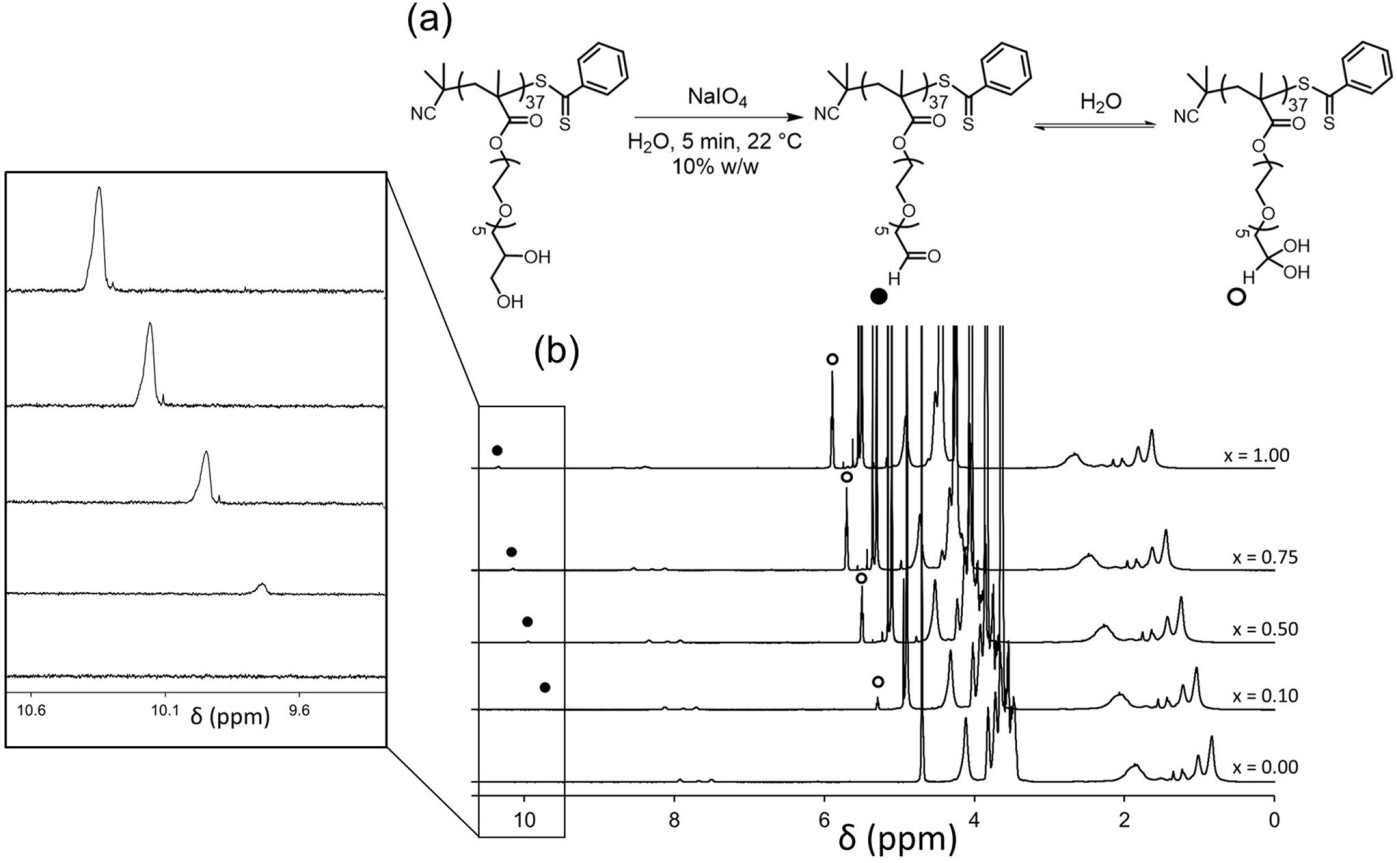
a) Reaction scheme for the (partial) oxidation of a near‐monodisperse PGEO5MA_37_ precursor in aqueous solution using NaIO_4_ at 22 °C. Adjusting the NaIO_4_/*cis*‐diol molar ratio (*x*) between 0.1 and 1.0 generates a library of aldehyde‐functional water‐soluble statistical copolymers. b) Offset ^1^H NMR spectra (D_2_O) recorded for PGEO5MA_37_, P(GEO5MA_n_‐stat‐AGEO5MA_m_)_37_ (where *m*=0.11, 0.49 and 0.78), and PAGEO5MA_37_.

Alternatively, RAFT aqueous solution polymerization of GEO5MA affords a near‐monodisperse PGEO5MA_37_ homopolymer (*M*_n_=17 200 g mol^−1^; *Đ*=1.18). When a NaIO_4_/*cis*‐diol molar ratio of unity was used to derivatize this precursor, essentially complete oxidation was achieved to afford PAGEO5MA_37_ homopolymer within 5 min at 22 °C (Table 1, Figure 3). The latter product proved to be water‐soluble at concentrations of up to 15 % w/w. In striking contrast, the product of the oxidation of PGMA_39_ homopolymer using a stoichiometric amount of periodate, denoted hereafter as PAGMA_39_, proved to be water‐insoluble when prepared at 1.5 to 10 % w/w (Supporting Information, Table S1). The much higher aqueous solubility observed for PAGEO5MA_37_ is attributed to the hydrophilic oligo(ethylene glycol) units on each repeat unit.

**Table 1:** Extent of oxidation, DMF GPC molecular weight and dispersity data for the selective oxidation of PGEO5MA_37_ in aqueous solution at 22 °C using (sub‐)stoichiometric NaIO_4_/*cis*‐diol molar ratios ranging between 0 and 1.0.

NaIO_4_/*cis*‐diol molar ratio	Extent of oxidation [%]	*M*_n_ [kg mol^−1^]	*Đ*
1.00	>99	16.5	1.22
0.75	78	15.9	1.24
0.50	49	16.8	1.21
0.10	11	17.4	1.22
0.00	0	17.2	1.18

However, only a minor fraction of monomer repeat units may need to be converted into aldehyde groups for certain applications. Thus, partial oxidation of a PGEO5MA_37_ precursor using sub‐stoichiometric quantities of NaIO_4_ oxidant relative to its *cis*‐diol groups was also investigated (schematic in Figure 3 a).

Accordingly, utilizing NaIO_4_/*cis*‐diol molar ratios of 0.10, 0.50 or 0.75 produced a series of water‐soluble P(GEO5MA_n_‐*stat*‐AGEO5MA_m_)_37_ statistical copolymers with approximate degrees of aldehyde functionality of 0.11, 0.49 and 0.78 respectively, as estimated from ^1^H NMR spectroscopy studies (Table 1, Figure 3). Thus, the target degree of aldehyde functionality is always achieved (within experimental error). DMF GPC analyses confirmed that neither partial nor full oxidation of the PGEO5MA_37_ homopolymer had a significant effect on its molecular weight distribution (Table 1; Figure S4). Moreover, using a slight excess of NaIO_4_ relative to the pendent *cis*‐diol groups also resulted in partial loss of the dithiobenzoate end‐groups. Similarly, a PGEO5MA homopolymer (*M*_n_=124.1 kg mol^−1^, *Đ*=4.55) was synthesized via free‐radical polymerization in aqueous solution at 70 °C for 18 h. Selective oxidation of the *cis*‐diol groups on this homopolymer also had minimal effect on its (broad) molecular weight distribution (Figures S5 and S6).

**Scheme 3:**
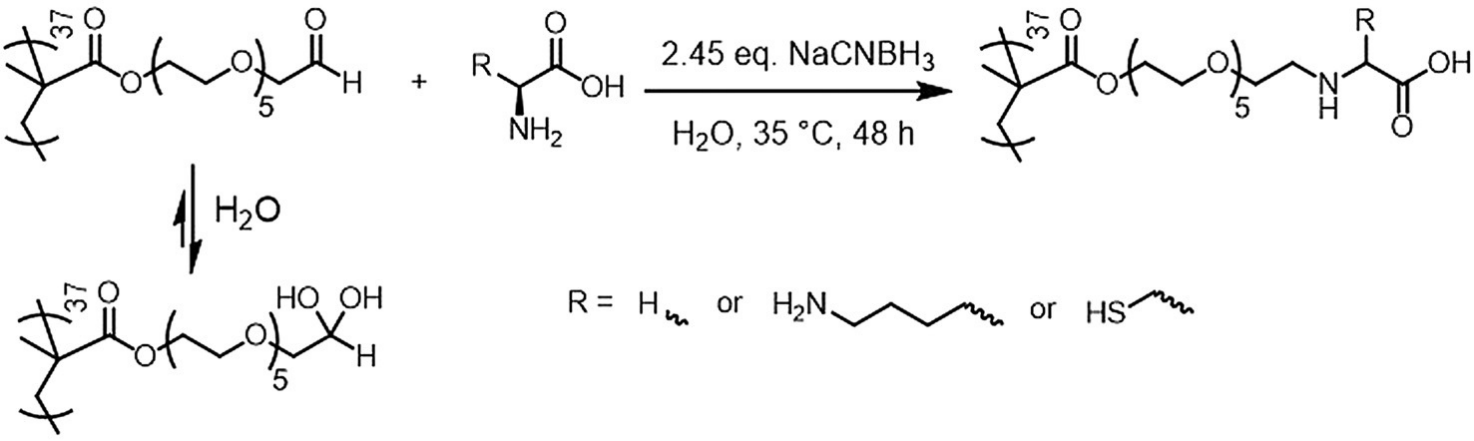
Schiff base reaction of PAGEO5MA_37_ with an amino acid (e.g., glycine, lysine, or cysteine) followed by reductive amination using excess aqueous NaCNBH_3_ at 35 °C to afford a series of new zwitterionic homopolymers via a two‐step one‐pot wholly aqueous protocol.

To investigate the scope of such new water‐soluble aldehyde‐functional polymers for conjugation with biologically‐relevant compounds, PAGEO5MA_37_ homopolymer was reacted in turn with three amino acids (glycine, lysine or cysteine; amino acid/aldehyde molar ratio=1.0) to form the corresponding Schiff base, followed by in situ reductive amination using excess NaCNBH_3_ (Scheme 3). These aqueous reaction mixtures were stirred at 35 °C for 48 h, with ^1^H NMR spectroscopy studies indicating very high extents of reaction (>99 %) in each case (Figure S7). Aqueous GPC analysis of the resulting water‐soluble polymers indicated that molecular weight distributions remained relatively narrow after this two‐step one‐pot derivatization (Figure S8).

**Scheme 4:**
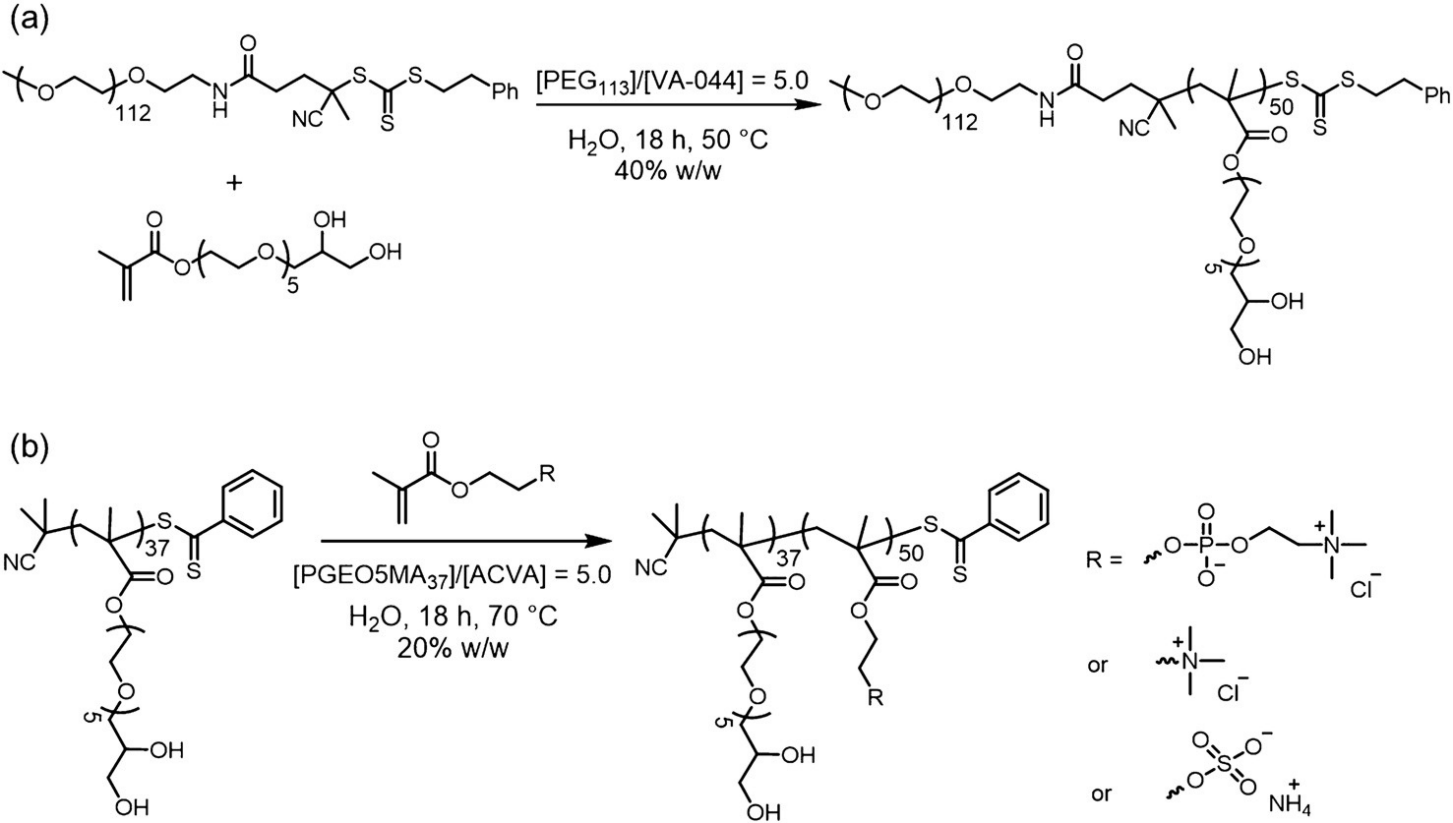
a) Reaction scheme for the synthesis of PEG_113_‐PGEO5MA_50_ via RAFT aqueous solution polymerization of GEO5MA at 40 % w/w solids using a PEG_113_/VA‐044 molar ratio of 5.0 at 50 °C. b) Reaction scheme for the synthesis of PGEO5MA_37_‐PX_50_ diblock copolymers (where X=MPC, METAC or SEM) at 20 % w/w solids using a PGEO5MA_37_/ACVA molar ratio of 5.0.

This protocol was then extended to water‐soluble diblock copolymers. A series of neutral, zwitterionic, cationic or anionic double‐hydrophilic diblock copolymers was prepared in which one of the blocks was PGEO5MA (Scheme 4). For the neutral diblock copolymer, a trithiocarbonate‐capped PEG_113_ precursor was simply chain‐extended via RAFT aqueous solution polymerization of GEO5MA at 50 °C. For the synthesis of the ionic diblock copolymers, a PGEO5MA_37_ precursor was chain‐extended via RAFT aqueous solution polymerization of 2‐(methacryloyloxy)ethyl phosphorylcholine (MPC), [2‐(methacryloyloxy)ethyl] trimethylammonium chloride (METAC) or ammonium 2‐sulfatoethyl methacrylate (SEM) at 70 °C. Each polymerization was allowed to proceed overnight to ensure high monomer conversion (≥98 % in all cases, as confirmed by ^1^H NMR spectroscopy; Table 2).

**Table 2:** Summary of monomer conversions, extents of *cis*‐diol oxidation and GPC molecular weight data for a series of neutral, zwitterionic, cationic and anionic diblock copolymers (with reference homopolymers included for comparison).

GPC eluent	Polymer composition	Monomer conversion [%]	Extent of *cis*‐diol oxidation [%]	*M*_n_ [kg mol^−1^]^[c]^	*Đ*
DMF	PEG_113_	–	–	5.0	1.13
DMF	PEG_113_‐PGEO5MA_37_	>99	–	27.7	1.20
DMF	PEG_113_‐PAGEO5MA_37_	–	>99	26.2	1.22
Aqueous^[a]^	PGEO5MA_37_	–	–	5.8	1.29
Aqueous^[a]^	PGEO5MA_37_‐PMPC_50_	>99	–	13.1	1.34
Aqueous^[a]^	PAGEO5MA_37_‐PMPC_50_	–	99	13.4	1.38
Aqueous^[b]^	PGEO5MA_37_	–	–	–	–
Aqueous^[b]^	PGEO5MA_37_‐PMETAC_50_	98	–	23.4	1.12
Aqueous^[b]^	PAGEO5MA_37_‐PMETAC_50_	–	>99	23.3	1.11
Aqueous^[a]^	PGEO5MA_37_	–	–	5.7	1.34
Aqueous^[a]^	PGEO5MA_37_‐PSEM_50_	>99	–	11.0	1.30
Aqueous^[a]^	PAGEO5MA_37_‐PSEM_50_	–	>99	12.7	1.36

[a] 0.2 M NaIO_3_, 0.05 M TRISMA buffer, pH 7. [b] 0.5 M acetic acid, 0.3 M NaH_2_PO_4_, pH 2. [c] Relative to PEG/PEO standards.

DMF GPC analysis indicated a high blocking efficiency for the RAFT solution polymerization of GEO5MA using the PEG_113_ macro‐CTA and the resulting PEG_113_‐PGEO5MA_50_ diblock copolymer had a relatively low dispersity (*Đ*=1.20; Table 2; Figure S9a). However, aqueous GPC analysis was required to assess the molecular weight distributions of the ionic diblock copolymers. (Table 2; Figures S9b–d). Oxidation of the pendent *cis*‐diol groups on the PGEO5MA_*x*_ chains was investigated using a NaIO_4_/*cis*‐diol molar ratio of unity at a diblock copolymer concentration of 20 % w/w. According to ^1^H NMR analysis, the extent of derivatization was at least 99 % in all cases (Table 2). DMF GPC analysis confirmed that periodate oxidation had minimal effect on the molecular weight distribution (*Đ*=1.22; Figure S9a) in the case of the PEG_113_‐PGEO5MA_50_ diblock copolymer. Similar results were obtained for the zwitterionic, cationic and anionic diblock copolymers when using aqueous GPC (Table 2; Figures S9b–d).

## Conclusion

In summary, we have reported the atom‐efficient synthesis of a new *cis*‐diol‐based methacrylic monomer (GEO5MA) that is readily converted into a hydrophilic aldehyde‐functional monomer (AGEO5MA) via selective oxidation using NaIO_4_ in aqueous solution. Unlike almost all other literature examples of aldehyde‐based vinyl monomers, this latter monomer is water‐soluble and can be polymerized with good control via RAFT aqueous solution polymerization. Alternatively, homopolymerization of the GEO5MA precursor under similar conditions affords a well‐defined water‐soluble PGEO5MA precursor that can be converted into PAGEO5MA under mild conditions using a stoichiometric amount of NaIO_4_ oxidant. On the other hand, using sub‐stoichiometric quantities of NaIO_4_ relative to the pendent *cis*‐diol units produces a range of water‐soluble aldehyde‐functional statistical copolymers. New PAGEO5MA‐based double‐hydrophilic diblock copolymers can be prepared and model Schiff base reactions have been conducted in aqueous solution under mild conditions using various amino acids to introduce zwitterionic groups. We anticipate that this new hydrophilic aldehydic vinyl monomer and its corresponding copolymers will offer a range of potential applications in the fields of cell biology and biomaterials.

## Conflict of interest

The authors declare no conflict of interest.

## Supporting information

As a service to our authors and readers, this journal provides supporting information supplied by the authors. Such materials are peer reviewed and may be re‐organized for online delivery, but are not copy‐edited or typeset. Technical support issues arising from supporting information (other than missing files) should be addressed to the authors.

Supplementary

## References

[1] M. B. Smith , J. March , March's Advanced Organic Chemistry: Reactions, Mechanisms, and Structure, Wiley, Hoboken, 2007.

[2] J. Collins , S. J. Wallis , A. Simula , M. R. Whittaker , M. P. McIntosh , P. Wilson , T. P. Davis , D. M. Haddleton , K. Kempe , Macromol. Rapid Commun. 2017, 38, 1600534. 10.1002/marc.201600534 27859945

[3] J. Collins , K. Kempe , P. Wilson , C. A. Blindauer , M. P. McIntosh , T. P. Davis , M. R. Whittaker , D. M. Haddleton , Biomacromolecules 2016, 17, 2755–2766. 27419537 10.1021/acs.biomac.6b00919

[4] L. Tao , G. Mantovani , F. Lecolley , D. M. Haddleton , J. Am. Chem. Soc. 2004, 126, 13220–13221. 15479065 10.1021/ja0456454

[5] C. Scholz , M. Iijima , Y. Nagasaki , K. Kataoka , Macromolecules 1995, 28, 7295–7297.

[6] Y. Nagasaki , T. Okada , C. Scholz , M. Iijima , M. Kato , K. Kataoka , Macromolecules 1998, 31, 1473–1479.

[7] M. Iijima , Y. Nagasaki , T. Okada , M. Kato , K. Kataoka , Macromolecules 1999, 32, 1140–1146.

[8] H. Otsuka , Y. Nagasaki , K. Kataoka , Biomacromolecules 2000, 1, 39–48. 11709841 10.1021/bm990005s

[9] T. Bilgic , H. A. Klok , Biomacromolecules 2015, 16, 3657–3665. 26441148 10.1021/acs.biomac.5b01116

[10] S. N. S. S. Alconcel , S. H. Kim , L. Tao , H. D. Maynard , Macromol. Rapid Commun. 2013, 34, 983–989. 23553922 10.1002/marc.201300205

[11] A. W. Jackson , D. A. Fulton , Macromolecules 2012, 45, 2699–2708.

[12] C. Cao , K. Yang , F. Wu , X. Wei , L. Lu , Y. Cai , Macromolecules 2010, 43, 9511–9521.

[13] D. E. Whitaker , C. S. Mahon , D. A. Fulton , Angew. Chem. Int. Ed. 2013, 52, 956–959; 10.1002/anie.201207953 23225748

[14] J. Huang , X. Chen , H. Qin , H. Liang , J. Lu , Polymer 2019, 160, 99–106.

[15] N. A. A. Rossi , Y. Zou , M. D. Scott , J. N. Kizhakkedathu , Macromolecules 2008, 41, 5272–5282.

[16] G. S. Heo , S. Cho , K. L. Wooley , Polym. Chem. 2014, 5, 3555–3558. 25580163 10.1039/C4PY00456F PMC4285359

[17] Z. Wu , H. Liang , J. Lu , Macromolecules 2010, 43, 5699–5705.

[18] N. Y. Xiao , A. L. Li , H. Liang , J. Lu , Macromolecules 2008, 41, 2374–2380.

[19] N. Xiao , H. Liang , J. Lu , Soft Matter 2011, 7, 10834–10840.

[20] B. S. Murray , D. A. Fulton , Macromolecules 2011, 44, 7242–7252.

[21] R. H. Wiley , P. H. Hobson , J. Polym. Sci. 1950, 5, 483–486.

[22] L. Qiu , C. R. Xu , F. Zhong , C. Y. Hong , C. Y. Pan , Macromol. Chem. Phys. 2016, 217, 1047–1056.

[23] W.-D. B. N.-Y. Xiao , L. Zhong , W.-J. Zhai , N. Y. Xiao , L. Zhong , W. J. Zhai , W. D. Bai , Acta Polym. Sin. 2012, 8, 818–824.

[24] M. E. Wechsler , H. K. H. J. Dang , S. D. Dahlhauser , S. P. Simmonds , J. F. Reuther , J. M. Wyse , A. N. Vandewalle , E. V. Anslyn , N. A. Peppas , Chem. Commun. 2020, 56, 6141. 10.1039/d0cc02200d PMC7377432 32364214

[25] M. Wu , J. Chen , W. Huang , B. Yan , Q. Peng , J. Liu , L. Chen , H. Zeng , Biomacromolecules 2020, 21, 2409–2420. 32310635 10.1021/acs.biomac.0c00347

[26] G. Sun , C. Cheng , K. L. Wooley , Macromolecules 2007, 40, 793–795. 19066633 10.1021/ma062592x PMC2597404

[27] G. Foyer , M. Barriol , C. Negrell , S. Caillol , G. David , B. Boutevin , Prog. Org. Coat. 2015, 84, 1–8.

[28] G. Sun , H. Fang , C. Cheng , P. Lu , K. Zang , A. V. Walker , J.-S. A. Taylor , K. L. Wooley , ACS Nano 2009, 3, 673–681. 19309173 10.1021/nn8007977 PMC2661032

[29] R. H. Wiley , P. H. Hobson , J. Polym. Sci. 1949, 4, 483–486.

[30] C. Legros , M. C. De Pauw-Gillet , K. C. Tam , S. Lecommandoux , D. Taton , Eur. Polym. J. 2015, 62, 322–330. 10.1039/c5sm00313j 25793873

[31] T. Ishizone , A. Hirao , S. Nakahama , T. Kakuchi , K. Yokota , K. Tsuda , Macromolecules 1991, 24, 5230–5231.

[32] J. Hwang , R. C. Li , H. D. Maynard , J. Controlled Release 2007, 122, 279–286. 10.1016/j.jconrel.2007.04.010 17599628

[33] M. Yokoyama , M. Miyauchi , N. Yamada , T. Okano , Y. Sakurai , K. Kataoka , S. Inoue , Cancer Res. 1990, 50, 1693–1700. 2306723

[34] E. M. Pelegri-O'Day , N. M. Matsumoto , K. Tamshen , E. D. Raftery , U. Y. Lau , H. D. Maynard , Bioconjugate Chem. 2018, 29, 3739–3745. 10.1021/acs.bioconjchem.8b00635 30358981

[35] R. M. Broyer , G. N. Grover , H. D. Maynard , Chem. Commun. 2011, 47, 2212–2226. 10.1039/c0cc04062b PMC3066092 21229146

[36] J. M. Stukel , R. C. Li , H. D. Maynard , M. R. Caplan , Biomacromolecules 2010, 11, 160–167. 19924844 10.1021/bm9010276

[37] K. L. Christman , H. D. Maynard , Langmuir 2005, 21, 8389–8393. 16114947 10.1021/la050646a

[38] K. L. Christman , M. V. Requa , V. D. Enriquez-Rios , S. C. Ward , K. A. Bradley , K. L. Turner , H. D. Maynard , Langmuir 2006, 22, 7444–7450. 16893251 10.1021/la0608213

[39] J. Blankenburg , K. Maciol , C. Hahn , H. Frey , Macromolecules 2019, 52, 1785–1793.

[40] R. J. Mancini , J. Lee , H. D. Maynard , J. Am. Chem. Soc. 2012, 134, 8474–8479. 22519420 10.1021/ja2120234

[41] E. Sawicki , T. R. Hauser , T. W. Stanley , W. Elbert , Anal. Chem. 1961, 33, 93–96.

[42] P. J. Flory , F. S. Leutner , J. Polym. Sci. 1948, 3, 880–890.

[43] H. W. Melville , P. R. Sewell , Makromol. Chem. 1959, 32, 139–152.

[44] H. E. Harris , J. G. Pritchard , J. Polym. Sci. Part A 1964, 2, 3673–3679.

[45] D. H. Williams , I. Fleming , Spectroscopic Methods in Organic Chemistry, McGraw-Hill, Oakland, 1995.

[46] R. Zhao , A. K. Y. Lee , R. Soong , A. J. Simpson , J. P. D. Abbatt , Atmos. Chem. Phys. 2013, 13, 5857–5872.

[47] M. Rivlin , U. Eliav , G. Navon , J. Phys. Chem. B 2015, 119, 4479–4487. 25742498 10.1021/jp513020y

[48] J. P. Lewicki , C. A. Fox , M. A. Worsley , Polymer 2015, 69, 45–51.

[49] A. Wolfel , M. R. Romero , C. I. Alvarez Igarzabal , Eur. Polym. J. 2019, 112, 389–399.

