# Supplementary material for: New Aldehyde‐Functional Methacrylic Water‐Soluble Polymers
Source: Angew Chem Int Ed Engl. 2021 May 3;60(21):12032–7. doi: 10.1002/anie.202015298 (PMC8252606; doi:10.1002/anie.202015298)
Supplement: Supplementary file 1 — Supplementary [file ANIE-60-12032-s001.pdf]

Angewandte Chemie
Eine Zeitschrift der Gesellschaft Deutscher Chemiker

Supporting Information

# New Aldehyde-Functional Methacrylic Water-Soluble Polymers

*Emma E. Brotherton, Craig P. Jesson, Nicholas J. Warren, Mark J. Smallridge, and Steven P. Armes**

anie_202015298_sm_miscellaneous_information.pdf

# Supporting Information for:

## Experimental

### Materials

All reagents were used as received unless otherwise stated. Methyl methacrylate (MMA), isopropylidene glycerol pentaethylene glycol ether, 4-methoxyphenol (MEHQ), titanate catalyst and ammonium 2-sulfatoethyl methacrylate (SEM, supplied as a 26% w/v aqueous solution) were provided by GEO Specialty Chemicals (Hythe, UK). 4,4'-Azobis(4-cyanopentanoic acid) (ACVA; > 98%), glycine (≥ 98%), lysine (≥ 98%), cysteine (97%), sodium periodate ($NaIO_4$, ≥ 99.8%), sodium cyanoborohydride ($NaCNBH_3$, 95%), sodium nitrate ($NaNO_3$, 99%), tris(hydroxymethyl)aminomethane (TRISMA, ≥ 99.8%), tris(hydroxymethyl)aminomethane hydrochloride (TRIS HCl, ≥ 99%), sodium dihydrogen phosphate ($NaH_2PO_4$, ≥ 99%), [2-(methacryloyloxy)ethyl] trimethylammonium chloride (METAC, supplied as an 80% w/w aqueous solution), $d_8$-toluene, and deuterium oxide ($D_2O$) were purchased from Sigma-Aldrich, UK.

2-Cyano-2-propyl dithiobenzoate (CPDB, >97%) was purchased from Strem Chemicals Ltd (Cambridge, UK). 2,2'-Azobis[2-(2-imidazolin-2-yl)propane]dihydrochloride (VA-044, ≥ 97%) was purchased from Wako Chemicals (Germany). 4-(((2-(Carboxyethyl)thio)carbonothioyl)thio)-4-cyano-pentanoic acid (CECPA, > 95%) was kindly provided by Boron Molecular (Melbourne, Australia). 2-(Methacryloyloxy)ethyl phosphorylcholine (MPC) was kindly provided by Biocompatibles (Farnham, UK). Dimethylformamide (DMF), dichloromethane and methanol were purchased from Fisher Scientific (UK).

## Methods

*$^{1}$H NMR spectroscopy.* Spectra were recorded in $D_2O$ using a 400 MHz Bruker Avance-400 spectrometer at 298 K with 16 scans being averaged per spectrum.

*$^{13}$C NMR spectroscopy.* Broadband $^{1}$H-decoupled $^{13}$C spectra were acquired at 100 MHz using a Bruker Avance-400 spectrometer operating at 298 K. All spectra were recorded in $D_2O$ with between 128 and 10 240 scans being averaged per spectrum.

*Attenuated Total Reflectance Fourier Transform Infrared (ATR-FTIR) Spectroscopy.* Spectra were recorded using a Perkin Elmer FTIR Spectrum Two equipped with UATR Two diamond with 32 scans being averaged per spectrum.

*DMF Gel Permeation Chromatography (GPC*). DMF GPC was used to determine the number-average molecular weights ($M_n$), weight-average molecular weights ($M_w$) and dispersities ($Đ$) of $PGEO5MA_{37}$, $PAGEO5MA_{37}$, $PAGEO5MA_{30}$ (obtained by homopolymerization of AGEO5MA), $PEG_{113}$ homopolymers, P(GEO5MA$_n$-*stat*-AGEO5MA$_m$)$_{37}$ copolymers and also the $PEG_{113}$-$PGEO5MA_{37}$ diblock copolymer. The instrument set-up comprised two Agilent PL gel 5 µm Mixed-C columns and a guard column connected in series to an Agilent 1260 Infinity GPC system operating at 60 °C. The GPC eluent was HPLC-grade DMF containing 10 mmol LiBr at a flow rate of 1.0 mL min$^{-1}$, the copolymer concentration was typically 1.0% w/w and calibration was achieved using either a series of ten near-monodisperse poly(methyl methacrylate) standards ranging from 1 080 g mol$^{-1}$ to 1 020 000 g mol$^{-1}$ or ten near-monodisperse poly(ethylene oxide) standards ranging from 3 800 g mol$^{-1}$ to 780 000 g mol$^{-1}$. Chromatograms were analyzed using Agilent GPC/SEC software.

*Aqueous Gel Permeation Chromatography (GPC).* $PGEO5MA_{37}$-$PMETAC_{50}$ and $PAGEO5MA_{37}$-$PMETAC_{50}$ diblock copolymers were analyzed at 0.5% w/w concentration in an acidic aqueous buffer comprising 0.50 M acetic acid and 0.30 M $NaH_2PO_4$ adjusted to pH 2 using concentrated HCl. The GPC set-up comprised an Agilent 1260 Infinity series degasser and pump, an 8 µm Agilent PL Aquagel-OH 30 column and a 5 µm Agilent PL Aquagel-OH 20 column. A series of ten near-monodisperse poly(ethylene oxide)

standards ranging from 3 800 g mol$^{-1}$ to 780 000 g mol$^{-1}$ were used for calibration. A refractive index detector was employed at 35 °C and the flow rate was 1.0 mL min$^{-1}$.

0.5% w/w PGEO5MA$_{37}$-PMPC$_{50}$ and PAGEO5MA$_{37}$-PMPC$_{50}$ diblock copolymers were analyzed using an eluent comprising 0.20 M $NaNO_3$ and 0.05 M TRISMA buffer adjusted to pH 7 using concentrated HCl. The GPC set-up comprised an Agilent 1260 Infinity series degasser and pump, an 8 µm Agilent PL Aquagel-OH 30 column and a 5 µm Agilent PL Aquagel-OH 20 column. A series of ten near-monodisperse poly(ethylene oxide) standards ranging from 3 800 g mol$^{-1}$ to 780 000 g mol$^{-1}$ were used for calibration. A refractive index detector was employed at 35 °C and the flow rate was 1.0 mL min$^{-1}$.

0.5% w/w PGEO5MA$_{37}$-PSEM$_{50}$ and PAGEO5MA$_{37}$-PSEM$_{50}$ diblock copolymers were analyzed using an eluent comprising 0.20 M $NaNO_3$ and 0.05 M TRISMA buffer adjusted to pH 7 using concentrated HCl. The GPC set-up comprised an Agilent 1260 Infinity series degasser and pump, an 8 µm Agilent PL Aquagel-OH 40 column and a 5 µm Agilent PL Aquagel-OH 30 column. A series of ten near-monodisperse poly(ethylene oxide) standards ranging from 3 800 g mol$^{-1}$ to 780 000 g mol$^{-1}$ were used for calibration. A refractive index detector was employed at 35 °C and the flow rate was 1.0 mL min$^{-1}$.

*Elemental microanalysis.* CHN contents were determined using an Elementar vario MICRO cube analyzer.

*Liquid Chromatography–Mass Spectrometry (LC-MS).* Mass spectrometry analysis was conducted using an Agilent 6530 Accurate-Mass Q-TOF instrument connected to an Agilent 1260 Infinity liquid chromatograph equipped with an Agilent extended C18 2.1 mm x 50 mm column. The mobile phase consisted of solvent A (0.1 % aqueous formic acid) and solvent B (acetonitrile with 0.1% formic acid) run as a gradient ranging from 5 % to 95 % solvent B over a 10 min period. The injection volume was 1.0 µL and the flow rate was 0.40 ml min$^{-1}$. Samples were analyzed using the electrospray ionization (ESI) positive mode unless otherwise stated.

*OH Number and Water Content.* OH numbers were determined by titration according to ASTM E 326 using a Mettler Toledo T70 Titroprocessor. Water contents were determined by titration according to ASTM E 203 using a Metrohm 787 KF Titrator.

*4-Methoxyphenol (MeHQ) Inhibitor Content.* The MeHQ content was determined according to ASTM D 3125 using a ThermoScientific Evolution 220 UV-visible spectrophotometer.

## Synthesis

*Transesterification of isopropylideneglycerol penta(ethylene glycol) ether to afford IPGEO5MA*

A 5 L transesterification rig equipped with an air sparge was charged with isopropylideneglycerol penta(ethylene glycol) ether (4.26 mol, 1500 g), MMA (22.97 mol, 2300 g) and MEHQ (10.4 mmol, 1.30

g). This reaction mixture was heated to 115 °C at a constant air sparge rate of 300 mL $min^{-1}$. Water/MMA distillate was periodically removed from the still head until the headspace reached a constant temperature (~100 °C) after 2 h. The reactor was periodically topped up with MMA to maintain a constant reaction volume. Titanate catalyst (2.0 g) was added by syringe and transesterification was allowed to proceed for 2 h under partial take-off conditions. At this point, the head temperature reached 95 °C and the reaction was switched to 'run and bump' conditions. Thus, the still head was allowed to fill up prior to draining in order to concentrate methanol within the distillate. The still head was drained every 20 min for 2 h, after which transesterification was deemed complete. The reaction mixture was allowed to cool overnight. The following day, MMA was stripped from the same rig at 80 °C under vacuum for 2 h. Deionized water (400 g) was added and allowed to react for 1 h at 80 °C in order to deactivate the titanate catalyst. The resulting precipitate was removed by filtration and the water was removed under vacuum at 80 °C for 2 h. The resulting colourless liquid (IPGEO5MA, 1500 g) had a water content of 0.01 % w/w and an OH number of 2.63 mg KOH $g^{-1}$. If full conversion had been achieved, an OH number of 0 mg KOH $g^{-1}$ would be expected in the absence of any water. Thus, the measured value indicates that high conversion was achieved for this transesterification reaction.

*Acidic Deprotection of IPGEO5MA*

IPGEO5MA (1500 g) was treated with an Ambersep 900 OH resin (150 g) to remove MEHQ inhibitor. The reaction solution was stirred for 20 min at 22 °C and the resin was removed by filtration. The residual level of MEHQ was determined to be 25 ppm using UV spectroscopy and this was subsequently topped up to 100 ppm by adding fresh MEHQ (0.92 mmol, 0.115 g). The IPGEO5MA was placed in a 3 L flask followed by addition of deionized water (300 g) and concentrated HCl (11 g). This reaction mixture was stirred for 48 h using a 300 mL $min^{-1}$ air sparge, while water was periodically topped up to maintain a constant reaction volume. The resulting homogeneous solution was neutralized using an ion exchange resin (150 g), which was then removed by filtration. Finally, water was removed from the reaction solution at 50 °C under vacuum for 4 h prior to analysis. The resulting yellowish liquid had a water content of 0.3 % w/w and an OH number of 289 mg KOH $g^{-1}$. If full conversion had been achieved, an OH number of 294 mg KOH $g^{-1}$ would be expected in the absence of any water. Thus, high conversion was achieved for this acid deprotection. Elemental microanalyses: C, 52.59; H, 8.66; N, 0.0%. $C_{17}H_{32}O_9$ requires C, 52.69; H, 8.22; N, 0.0%; FTIR (ATR $\nu_{max}$, $cm^{-1}$): 3442 (-OH, H-bonded), 2870 (C-H stretch), 1716 (C=O ester), 1637 (C=C stretch), 1453 ($-CH_3$), 1403 (-OH bending, alcohol), 1098 (C-O stretch, aliphatic ether); $^1$H NMR (400 MHz; $D_2O$), *J* values given in Hz, δ = 1.86 (3H, s, $CH_3C$), 3.39-3.78 (22H, m, $CH_2CH_2O$), 3.80 (1H, m, $CH_2CHOHCH_2OH$), 4.26 (2H, t, 4.5 Hz, $CH_2CH_2O$),

5.66 (1H, s, C$H_2$CH$_2$), 6.08 (1H, s, C$H_2$CH$_2$); $^{13}$C NMR (125 MHz, $D_2O$) δ = 159.56, 135.73, 127.04, 71.75, 70.33, 69.77, 68.63, 64.23, 62.60, 17.45; MS ESI positive m/z $(M+H)^+$ requires 381.4, found 381.2.

*Oxidation of GEO5MA using $NaIO_4$ and isolation of AGEO5MA monomer*

GEO5MA monomer (0.026 mol, 10 g) and $NaIO_4$ (0.026 mol, 5.62 g) were dissolved in water (150 g) and stirred in the dark at 22 °C. The extent of oxidation of the *cis*-diol group to afford an aldehyde (or hydrated geminal diol) group was determined to be ≥ 99% within 5 min by $^1$H NMR spectroscopy. The AGEO5MA monomer was purified by extraction with dichloromethane and excess solvent was removed under reduced pressure to give a yellowish viscous liquid (7.5 g, 82%). $^1$H NMR spectroscopy studies in $d_8$-toluene indicated that, after drying, the purified AGEO5MA monomer was predominantly in its aldehyde form (62%) with the remaining 38% corresponding to its hydrated geminal diol form. Elemental microanalyses: 62% $C_{16}H_{28}O_8$ + 38% $C_{16}H_{30}O_9$ requires C, 54.13%; H, 8.16%; N, 0.0%. Found: C, 53.97%; H, 7.98%; N, 0.0%. FTIR (ATR $\nu_{max}$, $cm^{-1}$): 3425 (-OH, H bonded), 2870 (C-H stretch), 1716 (C=O ester), 1637 (C=C stretch), 1453 (-$CH_3$), 1404 (-OH bending, alcohol), 1098 (C-O stretch, aliphatic ether); $^1$H NMR (400 MHz; $D_2O$), *J* values given in Hz, δ = 1.87 (3H, s, C$H_3$C), 3.43 (2H, d, *J* 4.5 Hz, C$H_2$C(OH)$_2$H), 3.59-3.69 (17H, m, C$H_2$CH$_2$O), 3.76 (2H, t, *J* 4.5 Hz, C$H_2$CH$_2$O), 4.27 (2H, t, 4.5 Hz, C(=O)OC$H_2$CH$_2$), 5.09 (1H, m, C*H*C(OH)$_2$), 5.67 (1H, s, C$H_2$CH$_2$), 6.09 (1H, s, C$H_2$CH$_2$), 9.52 (1H, s, C(=O)*H*); $^{13}$C NMR (125 MHz, $D_2O$) δ = 202.9, 169.4, 135.8, 126.9, 88.4, 73.5, 69.7, 68.7, 64.2, 17.5. MS ESI positive m/z $(M+Na)^+$ requires 389.4, found 389.2.

*Synthesis of PAGEO5MA$_{30}$ homopolymer by RAFT aqueous solution polymerization*

AGEO5MA monomer (4.31 mmol, 1.50 g), CECPA RAFT agent (0.14 mmol, 0.0441 g), VA-044 initiator (29 µmol, 0.0093 g; CECPA/VA-044 molar ratio = 5.0) and $H_2O$ (8.8 g) were weighed into a 30 mL sample vial. The reaction solution was degassed *via* $N_2$ purge for 30 min before placing the sample vial into an oil bath set at 50 °C for 5 h. The polymerization was quenched by removing the sample vial from the oil bath and exposing the reaction mixture to air. The AGEO5MA conversion was determined to be more than 99% by $^1$H NMR spectroscopy by comparing the integrated monomer vinyl signals at 5.69 and 6.11 ppm to the integrated signal assigned to the two oxymethylene protons attached to the ester group for the monomer (4.12 ppm) and polymer (4.38 ppm), respectively.

*Synthesis of PGEO5MA$_{37}$ homopolymer by RAFT solution polymerization in ethanol*

GEO5MA monomer (0.079 mol, 30.0 g), CPDB RAFT agent (1.97 mmol, 0.437 g) and ACVA initiator (0.395 mmol, 0.111 g; CPDB/ACVA molar ratio = 5.0) were weighed into a 100 mL round-bottomed flask. Ethanol (20 g) was added and the reaction solution was degassed *via* $N_2$ purge for 40 min. Then the reaction mixture was heated for 120 min by immersing the flask in an oil bath set at 70 °C. The polymerization was quenched by removing the flask from the oil bath and exposing the reaction mixture

to air. The GEO5MA conversion was 72% as determined by $^{1}$H NMR spectroscopy by comparing the integrated monomer vinyl signals at 5.66 and 6.14 ppm to the integrated signal assigned to the two oxymethylene protons attached to the ester group for the monomer (4.16 ppm) and polymer (4.31 ppm), respectively. The crude PGEO5MA was precipitated into excess diethyl ether to remove unreacted GEO5MA and any other impurities, filtered and redissolved in methanol. After reprecipitation into excess diethyl ether, the product was redissolved in water and freeze-dried to produce a viscous red liquid. The mean DP of the purified $PGEO5MA_{37}$ was determined to be 37 by end-group analysis using $^{1}$H NMR spectroscopy: the integrated five aromatic protons assigned to the dithiobenzoate chain-end at 7.34-8.03 ppm were compared to the two oxymethylene proton signals attached to the ester group at 4.02-4.34 ppm and also to the five methacrylic backbone signals at 0.78-2.71 ppm.

*Oxidation of $PGEO5MA_{37}$ homopolymer using $NaIO_4$*

The oxidation of $PGEO5MA_{37}$ using a $NaIO_4$/*cis*-diol molar ratio of unity is representative of the general protocol. $PGEO5MA_{37}$ homopolymer (15.7 µmol, 0.22 g) was added to 2.0 mL of a 0.29 M aqueous solution of $NaIO_4$ to give a polymer concentration of 10% w/w. This reaction mixture was stirred for 30 min at 22 °C. The extent of oxidation was determined by $^{1}$H NMR spectroscopy by comparing the integrated proton signals assigned to the aldehyde proton at 9.51 ppm and the proton adjacent to the geminal diols at 5.13 ppm to that of the two oxymethylene protons attached to the ester group at 4.11 ppm. The resulting $PAGEO5MA_{37}$ solution was purified by dialysis against deionized water for two days (with three changes of water per day). Partial oxidation of $PGEO5MA_{37}$ was achieved in essentially the same way. However, a lower $NaIO_4$/*cis*-diol molar ratio was used in each case.

*Synthesis of a PGEO5MA homopolymer by free-radical polymerisation in aqueous solution*

GEO5MA monomer (1.31 mmol, 0.5 g), ACVA initiator (32.9 µmol, 0.0092 g) and water (10 g) were weighed into a 27 mL sample vial. The reaction solution was degassed *via* $N_2$ purge for 30 min. The reaction mixture was then heated for 18 h by immersing the sample vial in an oil bath set at 70 °C. The polymerization was quenched by removing the vial from the oil bath and exposing the reaction mixture to air. The GEO5MA conversion was more than 99% as determined by $^{1}$H NMR spectroscopy (the integrated monomer vinyl signals at 5.66 and 6.14 ppm were compared to the integrated signal assigned to the two oxymethylene protons attached to the ester group for the monomer (4.16 ppm) and polymer (4.31 ppm), respectively). Selective oxidation of this polydisperse homopolymer ($M_n$ = 124 100 g mol$^{-1}$; $Đ$ = 4.55, as indicated by DMF GPC analysis vs. a series of PMMA calibration standards) was achieved under the same conditions as those employed for the near-monodisperse $PGEO5MA_{37}$ homopolymer.

*Derivatization of $PAGEO5MA_{37}$ homopolymer with various amino acids*

The following protocol for the reductive amination of $PAGEO5MA_{37}$ with glycine is representative. $PAGEO5MA_{37}$ (1.00 g of a 10% w/w aqueous solution), glycine (0.31 mmol, 23.4 mg) and excess $NaCNBH_3$ (0.77 mmol, 48.1 mg) were weighed into a 15 mL sample vial. A glycine/aldehyde molar ratio of unity was employed in combination with a 2.45-fold excess of $NaCNBH_3$. The reaction mixture was stirred at 35 °C for 48 h to ensure full conversion of aldehyde to the corresponding secondary amine *via* the imine intermediate. The overall conversion was determined by $^1$H NMR spectroscopy by comparing the integrated residual geminal diol signal at 5.10 ppm with the five methacrylic backbone protons at 0.78-2.71 ppm. The resulting aqueous solution of glycine-functionalized $PAGEO5MA_{37}$ was purified by dialysis against deionized water for two days (with at least three water changes per day). Essentially the same protocol was used for cysteine (0.31 mmol, 37.8 mg) and lysine (0.31 mmol, 45.7 mg) instead of glycine.

*Synthesis of $PEG_{113}$-$PGEO5MA_{50}$ diblock copolymer via RAFT aqueous solution polymerization of GEO5MA*

A trithiocarbonate-capped $PEG_{113}$ macro-CTA (52.5 µmol, 0.281 g), GEO5MA monomer (2.62 mmol, 1.00 g), VA-044 initiator (10.5 µmol, 3.4 mg) and water (1.93 g) were weighed into a 15 mL sample vial. A macro-CTA/initiator ratio of 5.0 was employed and a copolymer concentration of 40% w/w was targeted. The reaction solution was degassed for 30 min before placing the sample vial into an oil bath set at 50 °C for 18 h. The polymerization was quenched by removing the vial from the oil bath and exposing the reaction mixture to air. The GEO5MA monomer conversion was determined to be more than 99% by $^1$H NMR spectroscopy (the integrated vinyl monomer signals at 5.66 and 6.08 ppm were compared to that of the two oxymethylene protons attached to the ester group in the GEO5MA repeat units at 4.26 ppm).

*Synthesis of $PGEO5MA_{37}$-$PX_{50}$ diblock copolymers via RAFT aqueous solution polymerization*

The synthesis of a $PGEO5MA_{37}$-$PMPC_{50}$ diblock copolymer is representative and was conducted as follows. $PGEO5MA_{37}$ macro-CTA (16.9 µmol, 0.241 g), MPC monomer (0.847 mmol, 0.25 g), ACVA initiator (4.23 µmol, 1.2 mg) and water (1.97 g) were weighed into a 15 mL sample vial. A total copolymer concentration of 20% w/w was targeted and a macro-CTA/initiator ratio of 4.0 was employed. The reaction solution was degassed for 30 min before placing the sample vial into an oil bath set at 70 °C for 18 h. The MPC conversion was determined to be more than 98% by $^1$H NMR spectroscopy (the integrated vinyl monomer signals at 6.56-6.11 ppm were compared to the methacrylic backbone signals at 0.31-2.46 ppm). The other two diblock copolymers were prepared by replacing the MPC monomer with either METAC (0.120 mmol, 0.32 g of an 80% w/w stock solution) or SEM (0.110 mmol, 0.96 g of a 26% w/w stock solution).

*Oxidation of $PEG_{113}$-$PGEO5MA_{37}$ and $PGEO5MA_{37}$-$PX_{50}$ diblock copolymers using $NaIO_4$*

The oxidation of the *cis*-diol units in $PEG_{113}$-$PGEO5MA_{50}$ is representative of the general protocol. A 40% w/w aqueous $PEG_{113}$-$PGEO5MA_{50}$ solution (2.60 g, 1.04 g dry weight $PEG_{113}$-$PGEO5MA_{37}$, 0.11 mmol) was added to 5.6 ml of a 0.38 M aqueous solution of $NaIO_4$. The resulting ~15% w/w copolymer solution was stirred at 22 °C for 30 min and the extent of oxidation was determined by $^1$H NMR spectroscopy (the integrated signals for the aldehyde proton at 9.51 ppm and the proton adjacent to the geminal diol at 5.13 ppm were compared to that for the signal at 4.11 ppm corresponding to two oxymethylene protons attached to the ester group). The resulting diblock copolymer solution was purified by dialysis against deionized water for two days (with three changes of water per day).

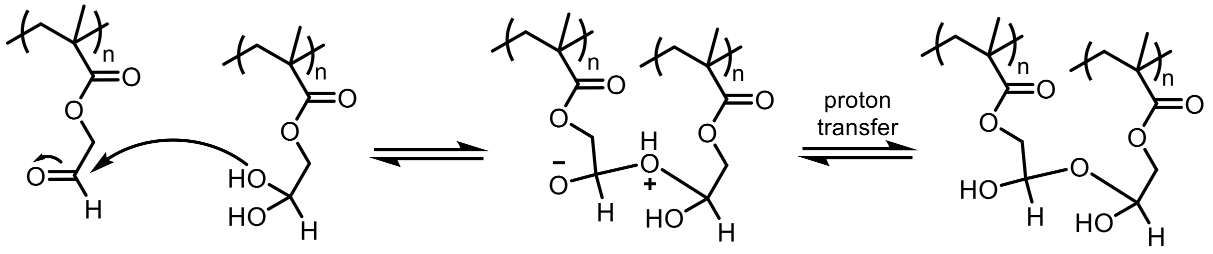


Scheme S1. Crosslinking side reaction that can occur between aldehyde and geminal diol groups on PAGMA.

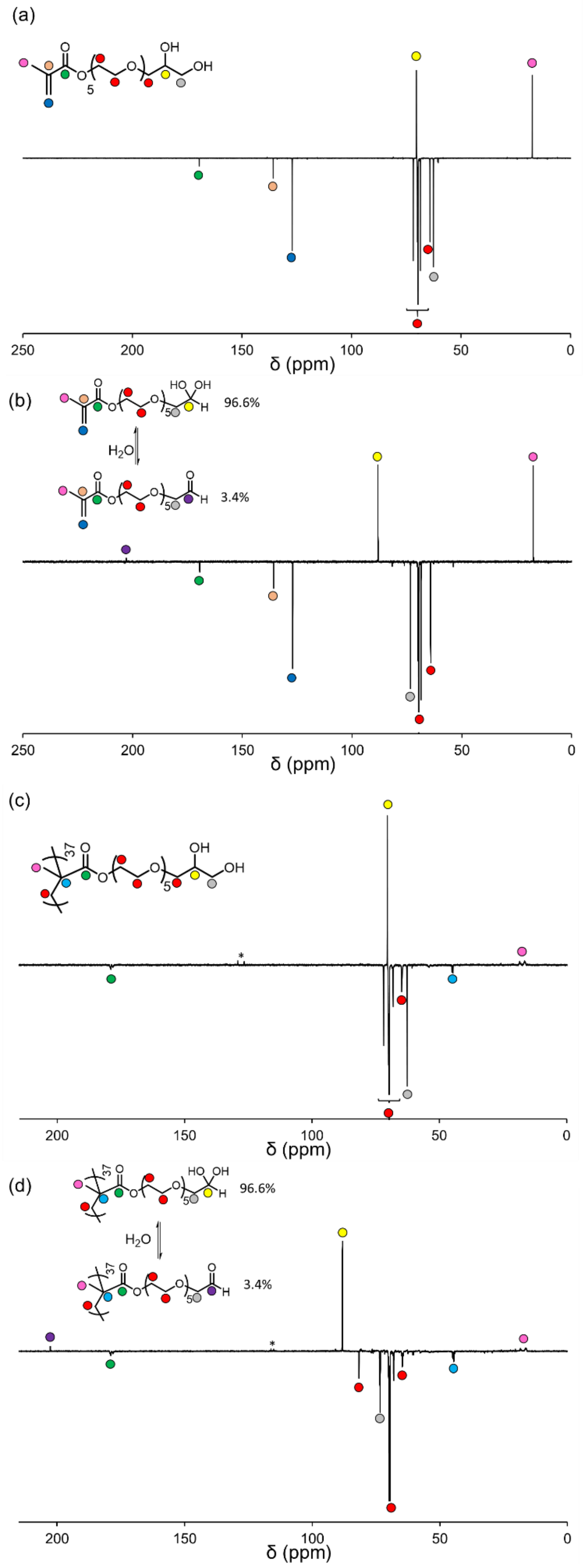


Figure S1. Assigned DEPTQ $^{13}$C NMR spectra ($D_2O$, 100 MHz) recorded for (a) GEO5MA monomer, (b) AGEO5MA monomer, (c) PGEO5MA$_{37}$ homopolymer and (d) PAGEO5MA$_{30}$ homopolymer (* indicate signals from RAFT chain ends).

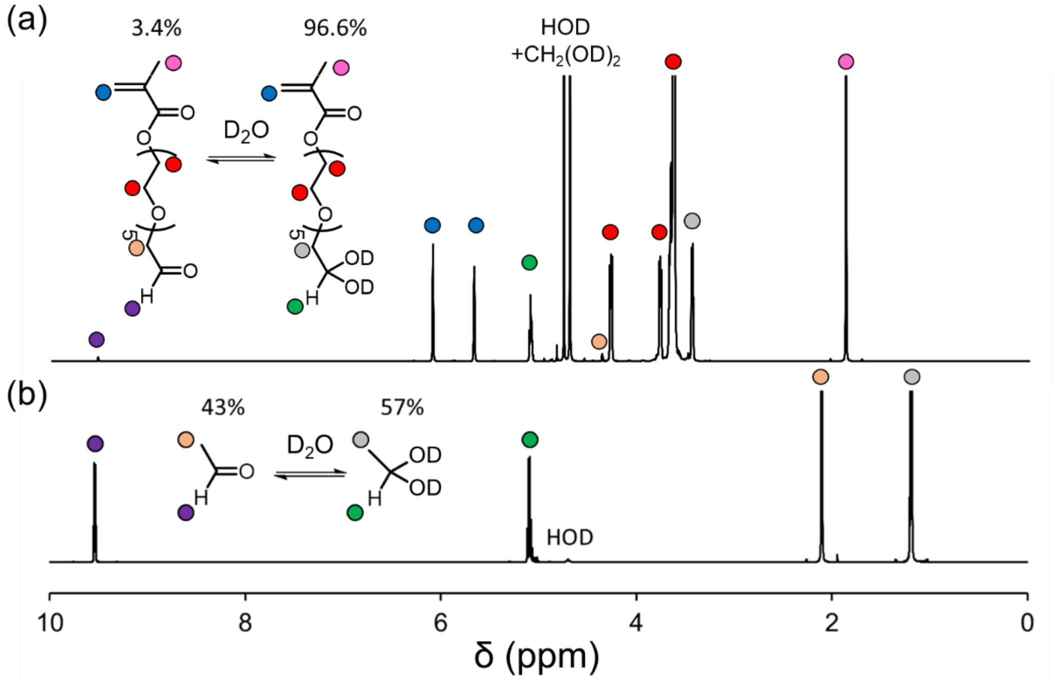


Figure S2. Assigned $^{1}$H NMR spectra ($D_2O$) recorded for (a) AGEO5MA monomer and (b) acetaldehyde (used as a reference compound). In the former case, the aldehyde group is mainly present in its hydrated germinal diol form.

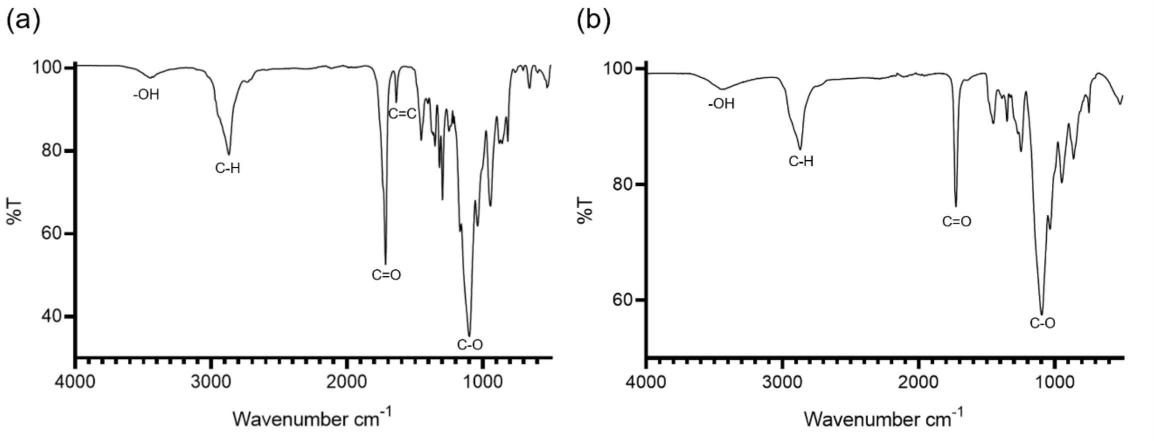


Figure S3. FT-IR spectra recorded at 22 °C for (a) AGEO5MA monomer and (b) PAGEO5MA homopolymer. [N.B. There is no detectable aldehyde carbonyl band in these spectra. This is because this group is mainly present in its hydrated germinal diol form. The relatively small number of aldehyde groups indicated by $^{1}$H NMR spectroscopy studies (see Figure 1b in the main manuscript) would only be expected to produce a very weak band, which is dominated by the strong *methacrylic ester* band that absorbs at a very similar wavenumber (1716 $cm^{-1}$)].

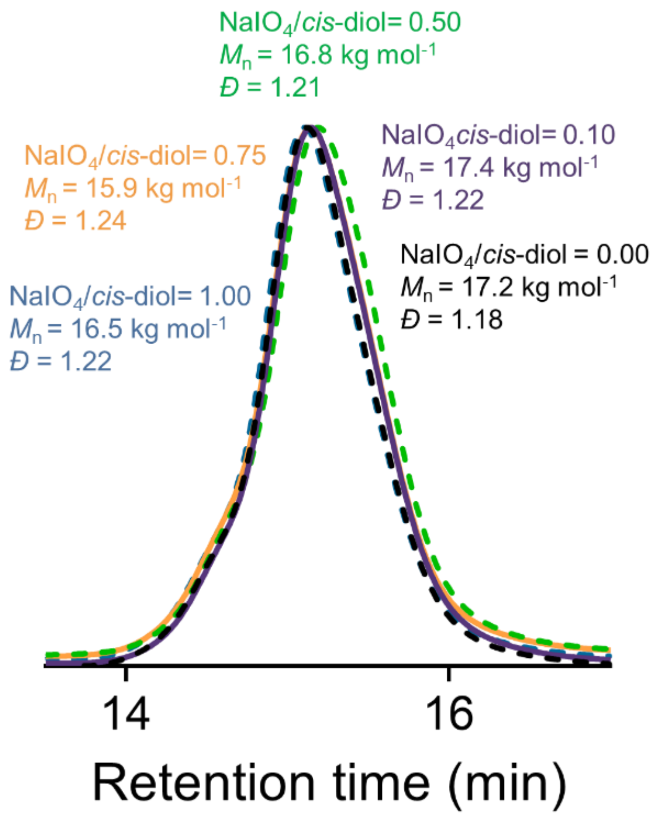


Figure S4. DMF GPC traces (vs. PMMA calibration standards) recorded for a $PGEO5MA_{37}$ precursor, $P(GEO5MA_n$-*stat*-$AGEO5MA_m)_{37}$ statistical copolymers (where m = 0.11, 0.49 and 0.78, when using a $NaIO_4$/*cis*-diol molar ratio of 0.10, 0.50 or 0.75, respectively) and $PAGEO5MA_{37}$ ($NaIO_4$/*cis*-diol molar ratio = 1.00). The desired degree of aldehyde functionality is achieved (within experimental error) when using sub-stoichiometric amounts of periodate and such selective oxidation has essentially no effect on the molecular weight distribution curve of the $PGEO5MA_{37}$ precursor.

Table S1. Summary of the aqueous solubilities observed for $PAGEO5MA_{37}$ and $PAGMA_{39}$ prepared at various concentrations.

| **$PAGEO5MA_{37}$ concentration** | | **$PAGMA_{39}$ concentration** | | **Water soluble?** | |
|---|---|---|---|---|---|
| % w/w | mol dm$^{-3}$ | % w/w | mol dm$^{-3}$ | $PAGEO5MA_{37}$ | $PAGMA_{39}$ |
| 1.5 | 0.0012 | 1.5 | 0.0027 | Soluble | Insoluble |
| 2.0 | 0.0016 | 2.0 | 0.0036 | Soluble | Insoluble |
| 3.5 | 0.0027 | - | - | Soluble | - |
| 10 | 0.0079 | 10 | 0.0196 | Soluble | Insoluble |
| 15 | 0.0118 | - | - | Insoluble | - |

All experiments were conducted at 22 °C using a $NaIO_4$/*cis*-diol molar ratio of unity.

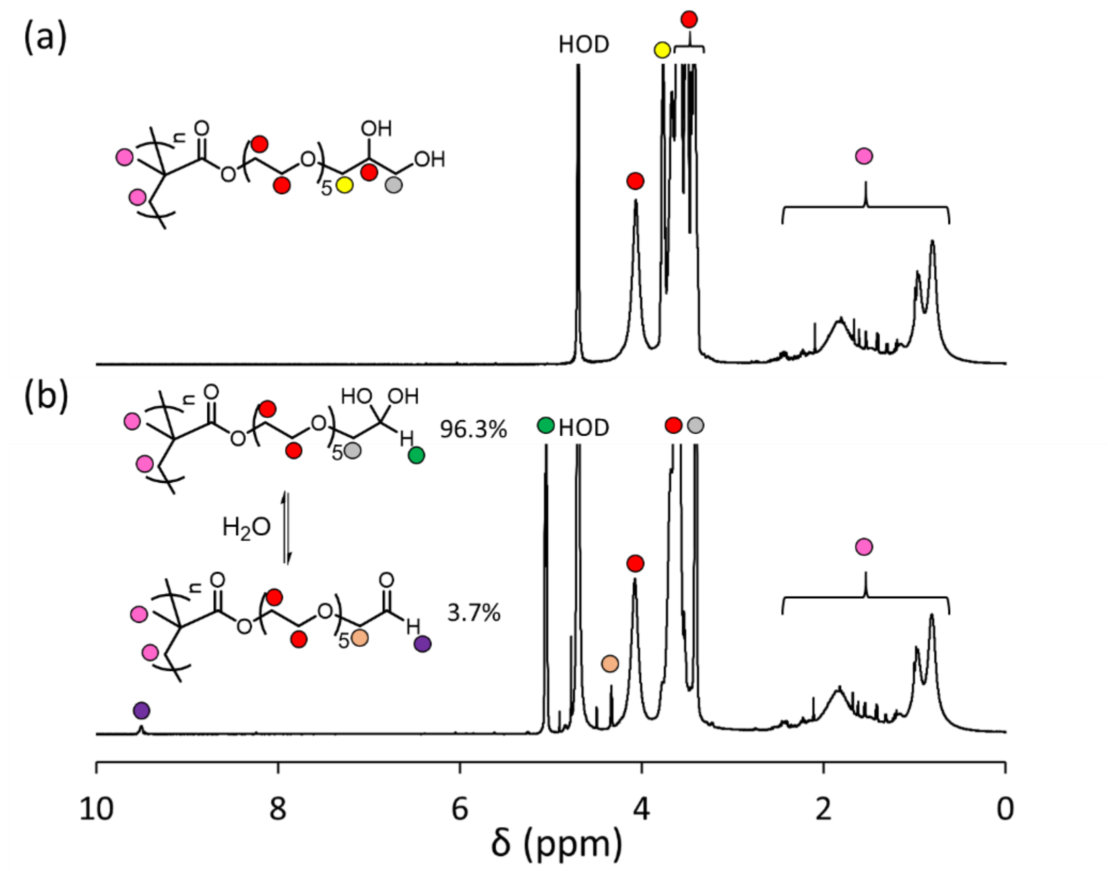


Figure S5. Assigned $^{1}$H NMR spectra ($D_2O$) recorded for (a) PGEO5MA synthesized by free-radical polymerisation and (b) PAGEO5MA synthesized by oxidising PGEO5MA using $NaIO_4$.

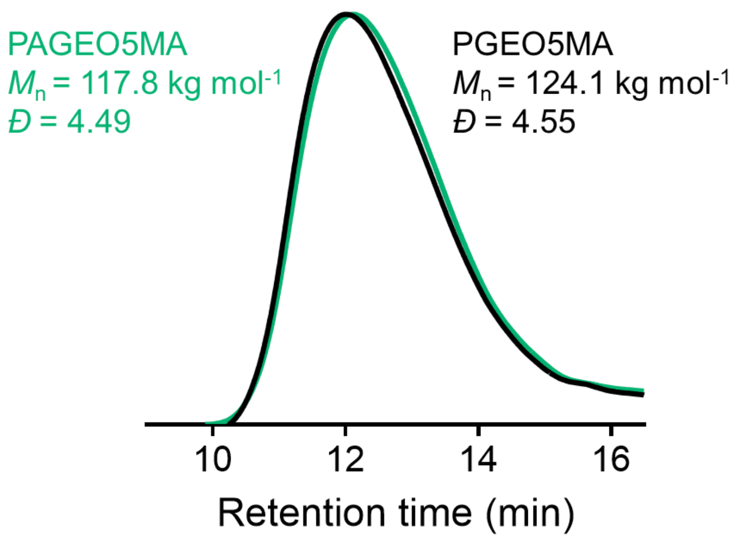


Figure S6. DMF GPC traces (vs. PMMA standards) recorded for PGEO5MA and PAGEO5MA homopolymers synthesized *via* free-radical polymerization in water.

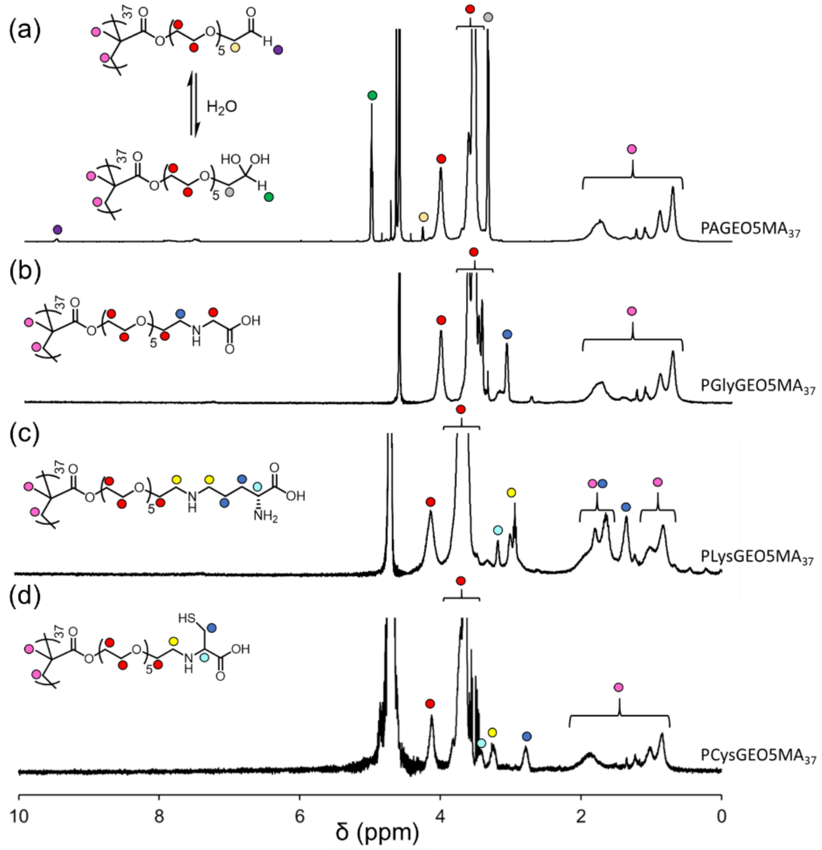


Figure S7. Assigned $^{1}$H NMR spectra ($D_2O$) recorded for (a) PAGEO5MA$_{37}$, (b) PGlyGEO5MA$_{37}$, (c) PLysGEO5MA$_{37}$ and (d) PCysGEO5MA$_{37}$.

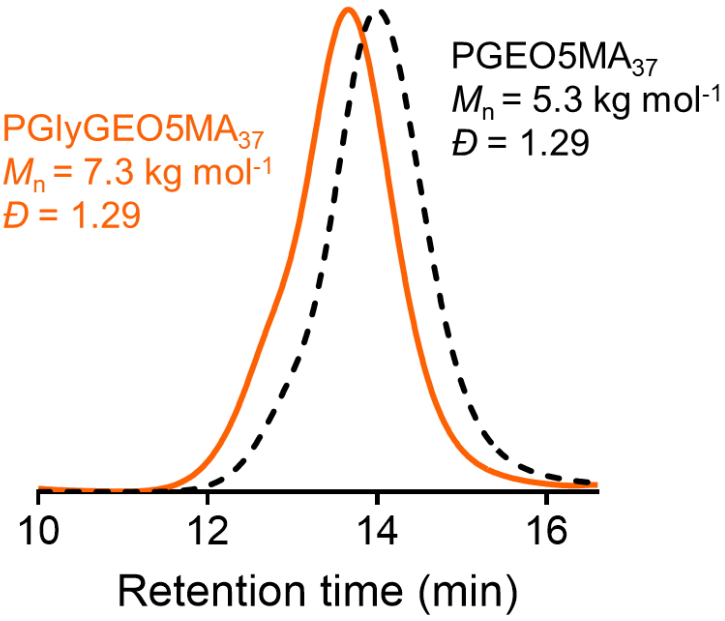


Figure S8. Aqueous GPC traces (vs. PEG/PEO calibration standards) recorded for PGEO5MA$_{37}$ and PGlyGEO5MA$_{37}$. After periodate oxidation, derivatization with glycine leads to a systematic shift in the molecular weight distribution curve but the dispersity (*Đ*) remains narrow.

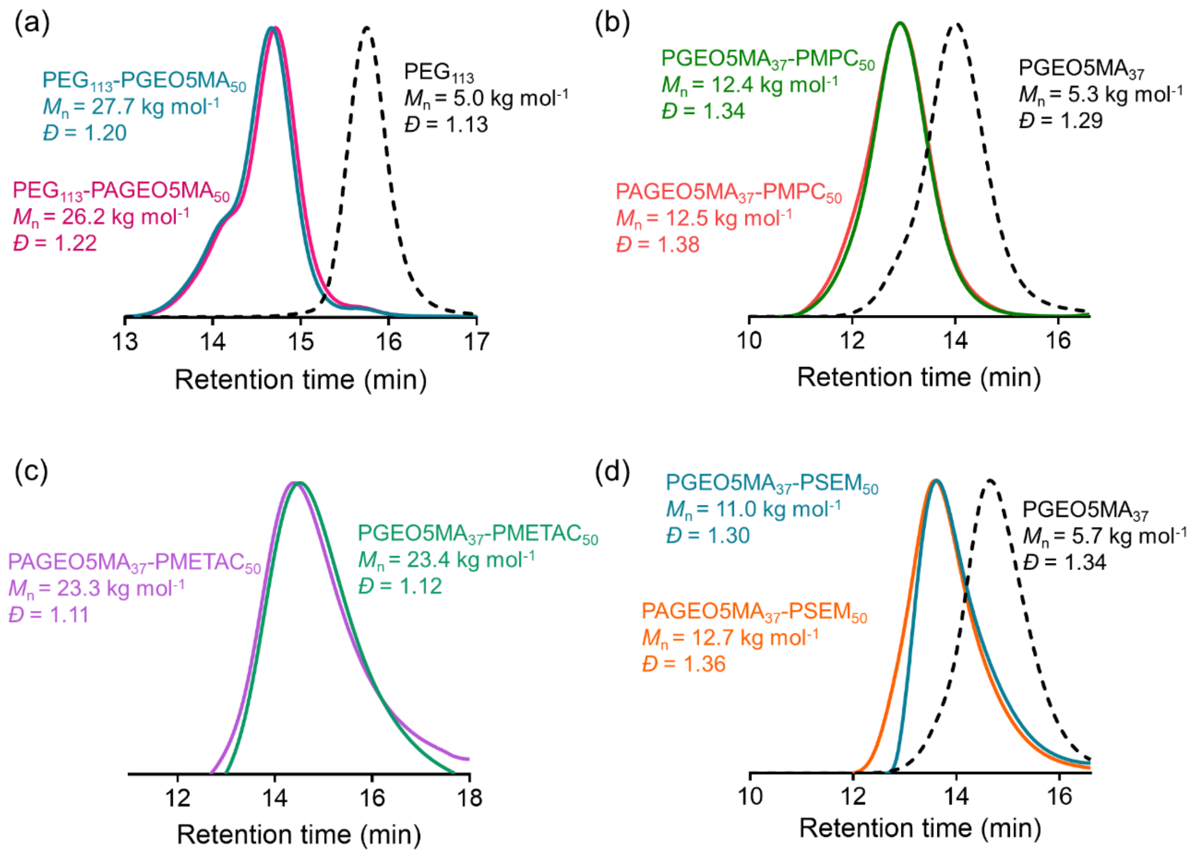


Figure S9. DMF GPC traces (vs. PEG/PEO calibration standards) recorded for (a) $PEG_{113}$-$PGEO5MA_{50}$, $PEG_{113}$-$PAGEO5MA_{50}$ and $PEG_{113}$ and aqueous GPC traces (vs. PEG/PEO calibration standards) recorded for (b) $PGEO5MA_{37}$-$PMPC_{50}$, $PAGEO5MA_{37}$-$PMPC_{50}$ and $PGEO5MA_{37}$, (c) $PGEO5MA_{37}$-$PMETAC_{50}$, $PAGEO5MA_{37}$-$PMETAC_{50}$ and (d) $PGEO5MA_{37}$-$PSEM_{50}$, $PAGEO5MA_{37}$-$PSEM_{50}$ and $PGEO5MA_{37}$. In the case of (a), (b) and (d), there is a substantial shift in molecular weight for the diblock copolymer relative to its corresponding homopolymer. Unfortunately, in (c) it was not possible to record the GPC curve for the $PGEO5MA_{37}$ precursor using this particular eluent (0.5 M acetic acid, 0.3 M $NaH_2PO_4$ pH 2). In all four cases, selective oxidation of the pendent *cis*-diol groups using sodium periodate had minimal effect on the molecular weight distribution.
